# Natural compounds based chemotherapeutic against Chagas disease and leishmaniasis: mitochondrion as a strategic target

**DOI:** 10.1590/0074-02760220396

**Published:** 2022-03-30

**Authors:** Danielle Lazarin-Bidóia, Francielle Pelegrin Garcia, Tânia Ueda-Nakamura, Sueli de Oliveira Silva, Celso Vataru Nakamura

**Affiliations:** 1 Universidade Estadual de Maringá, Laboratório de Inovação Tecnológica no Desenvolvimento de Fármacos e Cosméticos, Maringá, PR, Brasil

**Keywords:** trypanosomatids, natural products, mitochondrion, new therapeutic alternatives

## Abstract

Over the past years, natural products have been explored in order to find biological active substances to treat various diseases. Regarding their potential action against parasites such as trypanosomatids, specially *Trypanosoma cruzi* and *Leishmania* spp., much advance has been achieved. Extracts and purified molecules of several species from genera *Piper*, *Tanacetum*, *Porophyllum*, and *Copaifera* have been widely investigated by our research group and exhibited interesting antitrypanosomal and antileishmanial activities. These natural compounds affected different structures in parasites, and we believe that the mitochondrion is a strategic target to induce parasite death. Considering that these trypanosomatids have a unique mitochondrion, this cellular target has been extensively studied aiming to find more selective drugs, since the current treatment of these neglected tropical diseases has some challenges such as high toxicity and prolonged treatment time. Here, we summarise some results obtained with natural products from our research group and we further highlighted some strategies that must be considered to finally develop an effective chemotherapeutic agent against these parasites.

Chagas’ disease and leishmaniasis are neglected tropical and potentially lethal diseases that represent an important public health problem, especially in developing countries.^1^ Chagas disease, caused by the protozoan parasite *Trypanosoma cruzi*, is an infection that causes progressive damage to different organs, particularly the heart, esophagus, and lower intestine.^2^ It is considered one of the most important human parasitic infections in Latin America, affecting 6 to 7 million people with more than 10,000 deaths per year.^3^ In the last decades, due to the globalisation, this disease has been increasingly detected across Europe, United States, Africa, Canada, Eastern Mediterranean and Western Pacific countries.^3^ ^,^ ^4^ Leishmaniasis, a generic term for diverse clinical manifestations including cutaneous, mucocutaneous, and visceral disorders, is caused by protozoa parasites of more than 20 species of the genus *Leishmania*.^2^ It is considered an endemic disease in 98 countries or territories worldwide; it affects about 12 million people and it is estimated that there are 700,000 to 1 million new cases and 26 to 65 thousand deaths every year.^5^ ^,^ ^6^

Although Chagas’ disease was discovered more than 100 years ago,^7^ the available treatments are still restricted to only two nitro-derivative compounds, benznidazole and nifurtimox. Both compounds present important drawbacks, including serious side effects, long-term treatment, selective drug sensitivity on different *T. cruzi* strains, and non-effectiveness in the chronic phase of the disease.^8^ For leishmaniasis, the conventional therapies available are the pentavalent antimonials, amphotericin B, and miltefosine. The high cost, difficult administration, prolonged treatment time, and serious side effects caused by these therapies result in the early abandonment of treatment, which has led to the appearance of resistant parasite strains.^9^

Thus, the search for new therapeutic alternatives, less toxic and more effective, is essential for the treatment of patients with diseases caused by these trypanosomatids.^2^ Natural products have been a source of new and powerful drugs against several widespread diseases. In recent decades, bioactive phytocompounds present in the crude extracts and essential oils of medicinal plants have proven to be a good source of therapeutic agents, with several biological properties including antibacterial, antiprotozoal, antimycobacterial, antileishmanial, antitumor, and anti- human immunodeficiency virus (HIV).^10^ ^-^ ^17^ In addition, with advances in technology, large numbers of natural compounds from animals, marine organisms, and free-living or symbiotic microorganisms have also been investigated in the search of new drugs.^17^ ^,^ ^18^ ^,^ ^19^ It is believed that the vast Brazilian flora may be the basis for discovering new compounds that enable the production of innovative activities, including novel chemotherapeutics.^20^

The mitochondrion of trypanosomatids

The difficulty in developing more effective and selective compounds against *T. cruzi* and *Leishmania* spp. can be explained by the complex life cycles and distinct morphological and functional forms of these parasites. These parasites have special cytoplasmic structures, exclusive organelles and unique metabolic pathways in order to respond to the needs inherent to their specific lifestyle within their hosts. These peculiar features have been pointed as potential targets for the development of new drugs.^21^

The mitochondrion, the ‘‘master of life and death’’, is one of the most fascinating organelles of vital importance to survival of trypanosomatids. Unlike mammals, the protozoa *T. cruzi* and *Leishmania* spp. own a single mitochondrion that branches throughout the body of the parasite and comprises approximately about 12% of the cell.^21^ Thus, the proper function of the single mitochondrion in trypanosomatids is very important compared with mammalian cells that have several mitochondria because that ensures compensation for functionally impaired ones.^22^ Therefore, drugs that induce mitochondrial dysfunction in these parasites are considered potential candidates against these infections.^23^ Mitochondrion is a well-developed organelle that has a large number of mitochondrial ridges in all evolutionary forms of trypanosomatids, and it is involved in the production of energy; acts in cell death by apoptosis; in the production of heat and in the genetic contribution from mitochondrial DNA.^23^

Mitochondria also represent the main production site of reactive oxygen species (ROS) under physiological conditions, since different components of the respiratory chain can convert oxygen to superoxide anion radicals (O_2_•−) when reduced.^24^ ^,^ ^25^ The O_2_•− can be quickly dismuted to hydrogen peroxide (H_2_O_2_) by superoxide dismutases (SOD), and this, in the presence of Fe^2+^, generates the hydroxyl radical (HO•).^26^ ROS, at basal levels, play essential roles for cell physiology (i.e., cell signaling), however, high concentrations of these molecules induce oxidative stress, causing the oxidation of macromolecules, mainly lipids, proteins and DNA, which can trigger cell death.^27^ Then, to avoid cell damage caused by excess of ROS, mitochondria have antioxidant defense systems that eliminate these radicals by reducing them to water or alcohols.^28^

Trypanosomatids are devoid of glutathione reductase and thioredoxin reductase, present in many eukaryotic organisms, being responsible for the maintenance of intracellular redox homeostasis. Instead, trypanosomatids have an antioxidant system based on trypanothione [T(SH)_2_], responsible for the detoxification of hydroperoxides.^29^ ^,^ ^30^ T(SH)_2_ consists of two glutathione molecules covalently linked by a spermidine moiety and is maintained in its reduced form by the enzyme trypanothione reductase. This enzyme plays a central role in protection against reactive oxygen and nitrogen species through the recycling of oxidised trypanothione.^29^ ^,^ ^30^ This antioxidant defense system of trypanosomatids has been indicated as an attractive target for the development of new therapeutic actions.^31^ ^,^ ^32^ ^,^ ^33^

Activities of natural products against trypanosomatids

Our research group has been working with several plant extracts and some molecules isolated from them in the last 16 years, as summarised in Tables I-II. One of the first published works^34^ reported the results of a screening of extracts obtained from 19 species of plants used in Brazilian folk medicine for treatment of various diseases. The extracts were evaluated against axenic amastigote and promastigote forms of *L. amazonensis* and epimastigote forms of *T. cruzi*. Among those extracts, some presented interesting results inhibiting the growth of both parasites, such as hydroalcoholic extracts from *Cymbopogon citratus*, *Matricaria chamomilla*, *Piper regnellii*, *Tanacetum parthenium*, and *T. vulgare*. In addition, the extracts showed no cytotoxic effect on erythrocytes. These preliminary studies reinforced the necessity to deeply investigate those potential sources of new and selective agents for the treatment of these tropical diseases caused by protozoa.^34^ Some of those studies are described below.

**TABLE I:** Summary of major natural products evaluated by our research group against trypanosomatids

Species	Evaluated material	Activity					References
		*Trypanosoma cruzi*			*Leishmania amazonensis*			
		epimastigote	trypomastigote	amastigote	promastigote	amastigote		
*Achillea millefolium* *Anacardium occidentale* *Baccharis trimera* *Cymbopogon citratus* *Erythrina speciosa* *Eugenia uniflora* *Lippia alba* *Matricaria chamomilla* *Mikania glomerata* *Ocimum gratissimum* *Piper regnellii* *Prunus domestica* *Psidium guajava* *Punica granatum* *Sambucus canadensis* *Spilanthes acmella* *Stryphnodendron adstringens* *Tanacetum parthenium* *Tanacetum vulgare*	hydroalcoholic extract	39.8^a*^ 9.4^a*^ 65.8^a*^ 79.5^a*^ 26.0^a*^ 34.3^a*^ 36.5^a*^ 95.0^a*^ 49.5^a*^ 28.3^a*^ 89.7^a*^ 5.9^a*^ 15.6^a*^ 11.2^a*^ 13.8^a*^ 9.3^a*^ 51.9^a*^ 93.0^a*^ 95.5^a*^	- - - - - - - - - - - - - - - - - - **-**	- - - - - - - - - - - - - - - - - - **-**	11.6^a*^ 5.4^a*^ 58.3^a*^ 98.0^a*^ 5.1^a*^ 38.0^a*^ 8.5^a*^ 98.1^a*^ 52.5^a*^ 54.7^a*^ 98.2^a*^ 42.2^a*^ 65.4^a*^ 69.1^a*^ 65.7^a*^ 4.3^a*^ 36.5^a*^ 96.5^a*^ 96.4^a*^	48.2^a*^ 32.3^a*^ 64.6^a*^ 95.2^a*^ 28.4^a*^ 51.6^a*^ 57.5^a*^ 92.7^a*^ 97.5^a*^ 91.5^a*^ 96.8^a*^ 90.0^a*^ 52.0^a*^ 9.1^a*^ 54.9^a*^ 18.1^a*^ 21.0^a*^ 94.3^a*^ 99.0^a*^	^34^
*Piper regnellii*	eupomatenoid-3 eupomatenoid-5 eupomatenoid-6 conocarpan	26.3^b*^ 7.0^b*^ 7.5^b*^ 8.0^b*^	- - - -	- - - -	- - - -	- - - -	^35^
*Piper regnellii*	eupomatenoid-5	-	-	5.0^b+^	-	-	^36^
*Piper regnellii*	eupomatenoid-5	-	40.5^c#^	-	-	-	^37^
*Piper regnellii*	eupomatenoid-5	-	-	-	9.0^b+^	5.0^b$^	^38^
*Tanacetum parthenium*	aqueous extract ethanolic extract ethyl-acetate extract hexane fraction dichloromethane fraction ethyl-acetate fraction methanol fraction parthenolide	280.0^b*^ 3.0^b*^ 50.0^b*^ 2.1^b*^ 2.1^b*^ 79.0^b*^ 40.0^b*^ 0.5^b*^	- - - - - - - -	- - - - - - - 51.0^d*^	- - - - - - - -	- - - - - - - -	^40^
*Tanacetum parthenium*	guaianolide	18.1^c*^	5.7^c$^	66.6^c*^	-	-	^42^
*Tanacetum parthenium*	aqueous extract ethanolic extract hexane extract ethyl-acetate extract dichloromethane fraction hexane fraction ethyl-acetate fraction methanol fraction	- - - - - - - -	- - - - - - - -	- - - - - - - -	490.0^b&^ 434.0^b&^ 409.0^b&^ 29.0^b&^ 3.6^b&^ 7.0^b&^ 43.1^b&^ 43.8^b&^	74.8^b&^ 36.7^b&^ 42.4^b&^ <10^b&^ 2.7^b&^ 2.9^b&^ 28.3^b&^ 48.4^b&^	^43^
*Tanacetum parthenium*	parthenolide	-	-	-	0.37^b&^	0.81^b&^	^44^
*Porophyllum ruderale*	dichloromethane extract dichloromethane fraction compound A compound B	- - - -	- - - -	- - - -	60.3^b+^ 57.5^b+^ 7.7^b+^ 21.3^b+^	77.7^b+^ 72.5^b+^ 19.0^b+^ 28.7^b+^	^45^
*Arrabidaea chica*	hydroethanolic extract petroleum ether fraction hexane fraction chloroform fraction dichloromethane fraction ethyl-acetate fraction pheophorbide A	213.8^b*^ 689.0^b*^ 213.3^b*^ 204.7^b*^ 184.7^b*^ 556.7^b*^ >100^b*^	24.8^b$^ >100^b$^ 24.0^b$^ 19.8^b$^ 16.2^b$^ 66.7^b$^ 2.3^b$^	>100^b*^ - - 13.7^b*^ - - 2.3^b*^	- - - - - - -	- - - - - - -	^47^
*Copaifera reticulata*	copaiba oil	-	-	-	5.0^b+^	20.0^b+^	^51^
*Copaifera martii*	copaíba oil	-	-	-	-	100.0^e^	^53^
*Copaifera officinalis*	agathic acid hydroxycopalic acid kaurenoic acid methyl copalate pinifolic acid polyaltic acid	- - - - - -	- - - - - -	- - - - - -	28.0^b+^ 2.5^b+^ 28.0^b+^ 6.0^b+^ 70.0^b+^ 35.0^b+^	17.0^b+^ 18.0^b+^ 3.5^b+^ 14.0^b+^ 4.0^b+^ 15.0^b+^	^54^
*Copaifera spp.*	methyl copalate acid copalic acid 3β-hydroxycopalic acid agathic acid pinifolic acid polyaltic acid kaurenoic acid β-caryophylene	83.3^c*^ 42.7^c*^ 41.2^c*^ 86.8^c*^ 85.4^c*^ 167.7^c*^ 167.2^c*^ 78.4^c*^	377.3^c$^ 444.0^c$^ 453.1^c$^ 823.3^c$^ 163.0^c$^ 965.1^c$^ 596.0^c$^ 1,593.0^c$^	2.5^c*^ 1.3^c*^ 1.8^c*^ 14.9^c*^ 18.6^c*^ 28.4^c*^ 16.5^c*^ 63.7^c*^	- - - - - - - -	- - - - - - - -	^55^
*Copaifera reticulata* *Copaifera martii* *Copaifera cearenses* *Copaifera paupera* *Copaifera langsdorfii* *Copaifera multijuga* *Copaifera officinalis* *Copaifera lucens*	copaiba oil	17.0^b*^ 19.0^b*^ 35.0^b*^ 18.5^b*^ 30.5^b*^ 19.0^b*^ 21.5^b*^ 51.0^b*^	285.0^b$^ 97.5^b$^ 210.0^b$^ 182.5^b$^ 175.0^b$^ 242.5^b$^ 110.0^b$^ 215.0^b$^	<5.0^b*^ <5.0^b*^ 7.3^b*^ <5.0^b*^ 7.7^b*^ <5.0^b*^ <5.0^b*^ 10.0^b*^	- - - - - - - -	- - - - - - - -	^56^

Treatment time: (^#^) 2 h, (^$^) 24 h, (^&^) 48 h, (^+^) 72 h, (^*^) 96 h, (-) not evaluated; a: % growth inhibition in 100 μg/mL; b: concentration in μg/mL able to cause lysis or inhibit growth of 50% of the cells; c: concentration in μM able to cause lysis or inhibit growth of 50% of the cells; d: % growth inhibition in 2 μg/mL; e: reduction in the average lesion size of infected BALB/c mice.

**TABLE II:** Summary of alterations in trypanosomatids induced by natural products evaluated by our research group

Species	Protozoa	Alterations	References
Eupomatenoid-5 from *Piper regnellii*	*Trypanosoma cruzi* epimastigotes	SEM: rounded and deformed cells TEM: mitochondrial swelling, kinetoplast alteration, myelin-like figures, multinucleation	^36^
	*T. cruzi* amastigotes	TEM: cytoplasmic vacuolisation, autophagic vacuoles	
Eupomatenoid-5 from *Piper regnellii*	*T. cruzi* epimastigotes	FC: depolarisation of the mitochondrial membrane SA: lipid peroxidation, increased glucose-6-phosphate dehydrogenase, increase in H_2_O_2_ consumption	^37^
	*T. cruzi* trypomastigotes	SEM: plasma membrane damage with leakage of cytoplasmic contents TEM: plasma membrane detachment, cytoplasmic vacuolisation FC: depolarisation of the mitochondrial membrane SA: lipid peroxidation	
Eupomatenoid-5 from *Piper regnellii*	*T. cruzi* epimastigotes, trypomastigotes and amastigotes	FM: DNA fragmentation, autophagic vacuoles FC: depolarisation of the mitochondrial membrane, cell membrane disruption, increase in ROS/RNS production, phosphatidylserine exposure, decrease in cell volume SA: increase in mitochondrial O_2_•− production, decrease in total reduced thiol levels, lipid peroxidation	^32^
Eupomatenoid-5 from *Piper regnellii*	*Leishmania amazonensis* promastigotes	TEM: mitochondrial swelling, autophagic vacuoles, vesicles located in the flagellar pocket, myelin-like figures	^38^
Eupomatenoid-5 from *Piper regnellii*	*L. amazonensis* promastigotes	SEM: reduction and rounding of the cellular body FC: depolarisation of the mitochondrial membrane, phosphatidylserine exposure, decrease in cell volume, plasma membrane disruption, G0/G1 phase cell cycle arrest SA: increase in mitochondrial O_2_•− production, increased total ROS production, decrease in total reduced thiol levels	^39^
Parthenolide from *Tanacetum parthenium*	*T. cruzi* epimastigotes	SEM: distortion and decrease of the cell body and flagellum length TEM: mitochondrial swelling, multinuclear cells, increase of reservosomes, autophagic vacuoles, concentric structures, myelin-like figures, rearrangement of internal membranes	^40^
Parthenolide from *Tanacetum vulgare*	*T. cruzi* trypomastigotes	SEM: rounding and shortening of the parasite, loss of integrity of the plasma membrane	^41^
Guaianolide from *Tanacetum parthenium*	*T. cruzi* epimastigotes	SEM: thinning and stretching of the cell body and flagellum TEM: presence of membranes that involved organelles, myelin-like figures FC: cell volume reduction, decrease in mitochondrial membrane potential	^42^
	*T. cruzi* trypomastigotes	SEM: rounding and shortening of the parasite with leakage of cytoplasmic contents	
Parthenolide from *Tanacetum parthenium*	*L. amazonensis* promastigotes	TEM: vesicles located in the flagellar pocket, structures similar to large lysosomes	^44^
Compound A and B from *Porophyllum ruderale*	*L. amazonensis* promastigotes and amastigotes	SEM: rounded and swollen cells TEM: mitochondrial swelling FC: decrease in mitochondrial membrane potential	^46^
Pheophorbide A from *Arrabidaea chica*	*T. cruzi* trypomastigotes	SEM: rounding and shortening of the parasite body TEM: mitochondrial swelling, autophagic vacuoles, concentric membrane structures, abnormal chromatin distribution nuclear	^47^
	*T. cruzi* amastigotes	SEM: rounding of the parasite, plasma membrane damage	
Copaiba oil from *Copaifera reticulata*	*L. amazonensis* promastigotes	SEM: rounded shape with two flagella, protein denaturation of the cell surface TEM: mitochondrial swelling, vesicles located in the flagellar pocket, cytoplasmic vacuolisation	^52^
	*L. amazonensis* amastigotes	SEM: rupture of the plasma membrane with loss of their contents, protein denaturation of the cell surface TEM: mitochondrial swelling, vesicles located in the flagellar pocket, cytoplasmic vacuolisation FC: decrease in mitochondrial membrane potential, increase in permeability of the plasma membrane	
Hydroxycopalic acid from *Copaifera officinalis*	*L. amazonensis* promastigotes	SEM: rounded cells, rupture of the plasma membrane with loss of the cell contents, alterations of the flagellar membrane TEM: mitochondrial damage, vesicles located in the flagellar pocket, abnormal chromatin condensation nuclear	^54^
Agathic, kaurenoic and pinifolic acids from *Copaifera officinalis*	*L. amazonensis* amastigotes	FC: depolarisation of the mitochondrial membrane potential, increase in plasma membrane permeability	^54^
Terpenes from *Copaifera* spp.	*T. cruzi* epimastigotes	TEM: mitochondrial swelling, organelle disorganisation, membranous vacuole formation, concentric membrane structures FC: depolarisation of the mitochondrial potential, increase in plasma membrane permeability AS: lipid peroxidation	^55^
Copaiba oil from *Copaifera* spp.	*T. cruzi* epimastigotes	FC: depolarisation of the mitochondrial potential AS: lipid peroxidation	^56^
	*T. cruzi* amastigotes	TEM: swelling of the kinetoplast and chromatin condensation in both the nucleus and mitochondrion, disorganisation of the membranes	

SEM: scanning electron microscopy; TEM: transmission electron microscopy; FM: fluorescence microscopy; FC: flow cytometry; AS: spectrophotometric assay.

From the leaves of *P. regnellii* var. *pallescens*, a crude extract ethyl-acetate phase was obtained, then fractionated and eupomatenoid-3, eupomatenoid-5, eupomatenoid-6, and conocarpan were purified.^35^ Eupomatenoid-5 was the most active compound, exhibiting an interesting activity against epimastigotes,^35^ amastigotes,^36^ and trypomastigotes^37^ of *T cruzi*. This compound caused no lysis of the red blood cells and was more selective against the parasites than the Vero cells.^35^ Furthermore, eupomatenoid-5 at 7.0 μg/mL induced ultrastructural changes in epimastigotes of *T cruzi*, such as intense cytoplasmic vacuolisation, presence of myelin-like figures, and mitochondrial damage. In amastigotes, the compound at 7.0 μg/mL induced a decrease of 77.5% in the number of parasites internalised by LLCMK_2_ cells. Additionally, intense cytoplasmic vacuolisation and autophagic vacuoles were observed in *T. cruzi* intracellular amastigotes after 72 h of incubation.^36^

The mechanism of action of eupomatenoid-5 on epimastigotes and trypomastigotes of *T. cruzi* was also investigated.^37^ It was demonstrated that this compound induced depolarisation of the mitochondrial membrane, lipid peroxidation and increased glucose-6-phosphate dehydrogenase activity in epimastigotes. The plasma membrane and mitochondrion were the structures that were most affected in trypomastigotes and epimastigotes, respectively. Thus, the trypanocidal action of eupomatenoid-5 could be associated with mitochondrial dysfunction and oxidative damage, which can trigger destructive effects on biological molecules of *T. cruzi*, leading to parasite death.^37^

Additional evidence of the trypanocidal action of eupomatenoid-5 was demonstrated by Lazarin-Bidoia et al.,^32^ to better characterise the biochemical and morphological alterations induced by this compound in the three parasitic forms of *T. cruzi*: epimastigotes, trypomastigotes and amastigotes. Eupomatenoid-5 induced oxidative imbalance in all the parasitic forms, mainly trypomastigotes. The mechanistic assumption is that eupomatenoid-5 decreased the activity of trypanothione reductase, leading to a relative increase in the formation of ROS, that triggered a mitochondrial depolarisation (loss of mitochondrial membrane potential) followed by an absolute increase in mitochondrial ROS and reactive nitrogen species (RNS) production through the electron transport chain. The increase in ROS/RNS induced oxidative damage, leading to *T. cruzi* death.^32^

In parallel, eupomatenoid-5 activity against *L. amazonensis* was also investigated.^38^ As detected for *T cruzi*, it presented antileishmanial activity and selectivity after 72 h of treatment, exhibiting an IC_50_ of 9.0 μg/mL and 5.0 μg/mL for promastigotes and intracellular amastigotes, respectively. Transmission electron microscopy revealed several ultrastructural alterations, especially in the mitochondrion of treated parasites, indicating damage and significant change in this organelle (Fig. 1). These findings were deeply investigated by Garcia et al.^39^ It was shown that eupomatenoid-5 induced apoptosis-like cell death in *L. amazonensis* promastigotes due to the increased ROS production, decreased mitochondrial membrane potential, decreased total reduced thiol levels, phosphatidylserine exposure, decreased cell volume, and G0/G1 phase cell cycle arrest.^39^ It is important to note that both parasites treated with eupomatenoid-5, *T. cruzi* and *L. amazonensis*, showed mitochondrial damage (Fig. 1).

**Fig. 1:**
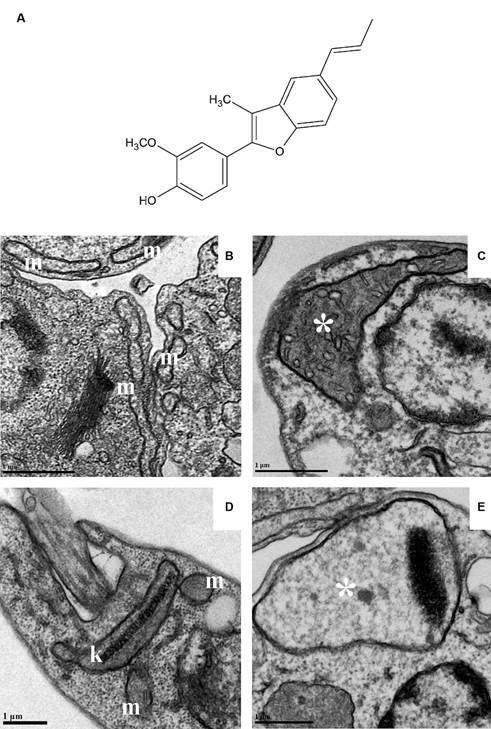
mitochondrial swelling in trypanosomatids after the treatment with eupomatenoid-5. (A) Chemical structure of eupomatenoid-5, the neolignan isolated from *Piper regnellii* var. *pallescens*. (B) Untreated epimastigotes of *Trypanosoma cruzi* presenting typical elongated morphology of the mitochondrion. (C) Epimastigotes treated with eupomatenoid-5 showing intense mitochondrial swelling. (D) Untreated promastigotes of *Leishmania amazonensis* presenting a typical mitochondrion with normal kinetoplast. (E) Promastigotes treated with eupomatenoid-5 showing remarkable mitochondrial swelling in the kinetoplast region. (m) mitochondrion; (k) kinetoplast; (*) mitochondrial swelling. Bars: 1 μm.

*Tanacetum* spp*.* (Asteraceae family) has also been extensively studied by our research group. Parthenolide is a sesquiterpene lactone that can be isolated from different species from the genus. In 2008,^40^ the crude extract, fractions and parthenolide obtained from *T. parthenium* showed to be active against epimastigotes and intracellular amastigotes of *T. cruzi*. The results evidenced a progressive increase in the antitrypanosomal effect during the purification process. Parthenolide showed an IC_50_ of 0.5 µg/mL for epimastigotes. At 2 µg/mL of parthenolide, the internalisation index of *T. cruzi* in LLMCK_2_ cells was reduced almost 51%. No hemolysis was detected for the pure compound at concentrations below 100 µg/mL. Further, parthenolide caused morphological and ultrastructural alterations in epimastigotes, including distortion of the cell body and decrease of the body and flagellum length, multinuclear cells, increase of reservosomes and autophagic vacuoles, formation of concentric structures resembling myelin-like figures, rearrangement of internal membranes, as well as mitochondrial swelling, which could be better seen in the kinetoplast region.^40^

Furthermore, it was demonstrated that the treatment with 1 µg/mL of parthenolide isolated from *T. vulgare* affected the morphology of *T. cruzi* trypomastigotes*,* inducing rounding and shortening of the parasite and loss of integrity of the plasma membrane.^41^ Moreover, parthenolide in combination with benznidazole exhibited a strong synergistic activity against epimastigotes, showing that the effect produced by a combination of components is greater than the sum of the effects produced by the components alone. On trypomastigotes, parthenolide in association with benznidazole displayed only an additive effect, (i.e., the combined effect of the two compounds was equal to the sum of the effect of each compound alone). Both compounds in combination exhibited a strong antagonism on LLCMK_2_ cells, resulting in greater protection of the cells from drug damage. Further studies are necessary to increase understanding of the mode of action of parthenolide alone or in combination with other drugs, for treatment of Chagas’ disease.^41^

Cogo et al.^42^ evaluated the *in vitro* activity of guaianolide (11,13-dehydrocompressanolide), another molecule isolated from *T. parthenium*. This compound was shown to be effective against *T. cruzi*, with IC_50_ values of 18.1 and 66.6 µM against epimastigote and amastigote forms, respectively. The best results were obtained against trypomastigotes, with an EC_50_ of 5.7 µM, showing also to be 16.4-fold more selective for the protozoan compared with mammalian cells (LLCMK_2_ cells). Further, a synergistic effect between guaianolide and benznidazole has been demonstrated for epimastigotes. Morphological and ultrastructural analysis of epimastigotes treated with guaianolide revealed a thinning and stretching of the cell body and flagellum, the presence of membranes that involved organelles and formation of myelin-like figures. Treated-trypomastigotes showed changes in body shape, with rounding and shortening of the parasite and apparent leakage of cytoplasmic contents. In addition, flow cytometry revealed a cell volume reduction and decrease in mitochondrial membrane potential in epimastigotes treated with guaianolide. The slight changes in mitochondrion observed in the parasites treated with guaianolide showed that this organelle is not the main target of this compound.^42^ However, this result differs from studies of sesquiterpene lactones, e.g. Izumi et al.,^40^ in which mitochondrial swelling was found.

Similarly, the biological activity of crude extracts and several fractions obtained from *T. parthenium* was investigated against *L. amazonensis*.^43^ A progressive increase in the antileishmanial effect was observed in the course of the purification process. In contrast, a progressive decrease in the cytotoxic effect against macrophages was observed in the course of the extraction process.^43^ Parthenolide has shown a significant activity against the promastigotes with IC_50_ of 0.37 µg/mL and for intracellular amastigotes, parthenolide reduced by 50% the survival index of parasites in macrophages when it was used at 0.81 µg/mL. The purified compound caused no cytotoxic effects against J774G8 macrophages and did not cause lysis in red blood cells. The cysteine protease activity increased following treatment of promastigotes with parthenolide and marked ultrastructural changes were detected by electron microscopy, such as structures similar to large lysosomes and increase of vesicles located in the flagellar pocket.^44^

Another class of natural compounds that was studied by our group was thiophene derivatives isolated from aerial parts of *Porophyllum ruderale,* a native plant from Brazil.^45^ It was shown that the crude extract presented activity against promastigote and axenic amastigote forms of *L. amazonensis* with an IC_50_ of 60.3 and 77.7 μg/mL, respectively. Then two thiophene derivatives were isolated: 5-methyl-2,2’:5’,2”-terthiophene (compound A) and 5’-methyl-[5-(4-acetoxy-1-butynyl)]- 2,2’-bithiophene (compound B). The activity of the purified compounds against promastigote and axenic amastigote forms was better than that of the crude extract and more selective against protozoa than for macrophage cells. The IC_50_ values of the compound A against promastigote and axenic amastigote forms were 7.7 and 19.0 μg/mL and for compound B these values were 21.3 and 28.7 μg/mL, respectively.^45^ Both compounds were used to further understand the mechanism of action involving parasite death.^46^ Promastigotes treated with compound A had a decreased mitochondrial membrane potential and this was confirmed by transmission electron microscopy as a mitochondrial swelling. Furthermore, promastigotes treated with compounds A and B showed rounded and swollen cells by scanning electron microscopy, confirming their biological potential activity against these parasites.^46^

Another interesting natural product that has been studied by our research group is pheophorbide A, purified from the leaves of *Arrabidaea chica*, known to be a photosensitiser that can be used in photodynamic therapy.^47^ This method of treatment is of great importance due to its selectivity. Pheophorbide A was significantly more effective in the presence of light, exhibiting greater activity against the clinically important trypomastigote and amastigote forms, with an EC_50_ of 2.3 μg/mL and IC_50_ of 2.3 μg/mL, respectively. It additionally caused significant morphological and ultrastructural changes in trypomastigotes and intracellular amastigotes. Treated-trypomastigotes presented alterations that were indicative of cell death through autophagic and apoptotic processes, including rounding and shortening of the parasite body, mitochondrial swelling, the presence of large vacuoles (e.g., autophagosomes) that occupied a large portion of the cytoplasm, the formation of concentric membrane structures in the cytosol, involving organelles and small vesicles, and nuclear alterations, such as abnormal chromatin distribution. Treated-amastigotes presented rounding of the parasite, and plasma membrane damage. Given the results, the pheophorbide A may have potential for use in blood banks for hemoprophylaxis, in which it presented significant efficacy against trypomastigotes and low toxicity in host cells.^47^

Finally, our group has been also focusing on one special natural product: the *Copaifera* oil, also called oleoresin. It is obtained from copaiba trees (Fabaceae family) which are present in several regions of Brazil. Oil resin extracted from this genus is one of the most important natural resources in traditional medicine for populations in the Amazon region, and it has been used by natives for several decades due to its medicinal properties.^48^ ^,^ ^49^ Its broad use is due to its healing and anti-inflammatory properties, widely described in the literature. Resin oil is mainly made up of sesquiterpenes and diterpenes, the former being the most abundant and responsible for the typical aroma of copaiba oil. Among the sesquiterpenes, we can mention β-caryophyllene, α-copaene, β-humulene, α-cubeben, and others. From diterpenes the most found are diterpenic acids, with copalic, hardwickic, covalenic and chlorequinic acids being the most reported in the literature.^50^

In addition to its healing and anti-inflammatory properties, copaiba oil and the chemical substances purified from *Copaifera* spp. have been studied for other medicinal purposes. In this sense, Santos et al.^51^ evaluated the effect of eight different Brazilian copaiba oils on *L. amazonensis*. Most of them presented IC_50_ values between 5.0 and 22.0 µg/mL. The most active oil was that from *C. reticulata* (collected in Pará State, Brazil) with IC_50_ of 5.0 and 20.0 µg/mL for promastigote and intracellular amastigote forms, respectively. Moreover, this copaiba oil showed low cytotoxicity against J774G8 macrophages.^51^ Given these results, copaiba oil from *C. reticulata* was used to investigate possible targets in promastigote and amastigote forms of *L. amazonensis*.^52^ The most prominent effect of the treatment was on mitochondrion, demonstrated by transmission electron microscopy and fluorimetric analysis. The copaiba oil treatment also induced cytoplasmic vacuolisation, increase of vesicles located in the flagellar pocket and loss of plasma membrane integrity.^52^

Similar results were observed after the treatment of promastigotes of *L. amazonensis* with copaiba oil obtained from *C. martii.* ^53^ By transmission electron microscopy, the mitochondrial swelling was also the most prominent effects observed in treated parasites. This was confirmed by post-evaluation of the mitochondrial membrane potential, which was decreased, indicating depolarisation of the mitochondrial membrane potential. Plasma membrane permeability was also altered. In addition, *in vivo* assay was performed in *L. amazonensis*-infected BALB/c mice, a murine model of cutaneous leishmaniasis. The oral treatment and oral plus topical treatment with copaiba oil caused significant reductions in the average lesion size compared with untreated mice, and showed no significant difference compared to the Glucantime-treated animals. Histopathological evaluation did not reveal changes in the organs of animals treated with copaiba oil. Mutagenicity evaluation (micronucleus test) showed no genotoxic effects in the copaiba oil-treated animals (*Mus musculus* (Swiss) outbred mice).^53^

Santos et al.^54^ further investigated specifically the antileishmanial activity of diterpene acids from the oleoresins isolated from *C. officinalis* (agathic, hydroxycopalic, kaurenoic, methyl copalate, pinifolic and polyaltic acids). Hydroxycopalic acid and methyl copalate were the most effective compounds against promastigotes (IC_50_ values of 2.5 and 6.0 μg/mL, respectively). However, pinifolic and kaurenoic acid showed to be more active against axenic amastigotes (IC_50_ of 3.5 and 4.0 μg/mL, respectively). Diterpene acids had low toxicity to mammalian erythrocytes. Promastigotes treated with hydroxycopalic acid showed morphological and ultrastructural changes, such as appearance of rounded cells, rupture of the plasma membrane with loss of the cellular contents, alterations of flagellar membrane, abnormal chromatin condensation, increase of vesicles located in the flagellar pocket, and mainly swollen mitochondrion with the appearance of concentric membrane structures inside the organelle. By flow cytometry, plasma membrane and mitochondrial membrane potential also showed to be significantly altered in axenic amastigotes after the treatment with agathic, kaurenoic and pinifolic acids.^54^ All our antileishmanial studies with copaiba oils (*C. reticulata*, *C. martii*, and *C. officinalis*) have often demonstrated alterations in the mitochondrial ultrastructure, which led to the loss of cell viability and confirmed the importance of this organelle for the parasite’s viability.^52^ ^,^ ^53^ ^,^ ^54^

Additionally, the biological properties of copaiba oils were also investigated against epimastigote, trypomastigote, and amastigote forms of *T. cruzi*.^55^ Seven acid diterpenes (methyl copalate, copalic acid, 3β-hydroxycopalic acid, agathic acid, pinifolic acid, polyaltic acid, kaurenoic acid), and one sesquiterpene (β-caryophylene) from *Copaifera* sp. were tested in order to investigate their activity and mechanism of action. Amastigotes were more sensitive to the presence of the compounds. Copalic acid was the most effective against amastigotes (IC_50_ of 1.3 μM). β-caryophylene was the least cytotoxic on LLCMK_2_ cells. Terpenes caused changes in the parasite ultrastructure, especially on mitochondrion. Alterations in the plasma membrane permeability, lipid peroxidation, and mitochondrial potential were also observed in parasites treated with the terpenes. In addition, the compounds were combined with each other in different concentrations to evaluate any possible synergic activity. β-caryophylene combined with copalic acid showed strong synergy, increasing in 40-fold the activity against trypomastigotes. This was the first study showing synergic activity between two terpenes against *T. cruzi*.^55^

Further, Izumi et al.^56^ compared the antitrypanosomal activity of copaiba oleoresins from eight different species of the genus *Copaifera*. It was shown that all oleoresins presented activity against all parasite life stages. *C. martii* and *C. officinalis* exhibit the best activities. The IC_50_ varied from >5.0 to 10.0 μg/mL for intracellular amastigotes, and the best values for epimastigotes and trypomastigotes were 17.0 μg/mL and 97.0 μg/mL, respectively. Additionally, oleoresins exhibited moderate cytotoxicity to LLCMK_2_ cells and low hemolytic effect. It was also observed lipid peroxidation, increased plasma membrane permeability and altered mitochondrial potential after treatment with many oleoresins. All of the treatments reduced the parasite dimensions and caused disorganisation of the membranes. *C. officinalis* caused swelling of the kinetoplast and chromatin condensation in both nucleus and mitochondrion.^56^ Considering all these results, copaiba oil has demonstrated its potential as an alternative possible new drug used against both trypanosomatid parasites.

Future perspectives

Considering the results from our studies, several natural compounds can affect mitochondrial function of trypanosomatids (Fig. 2). In fact, the interaction with the mitochondrion is very important due to the special properties of this organelle.

**Fig. 2:**
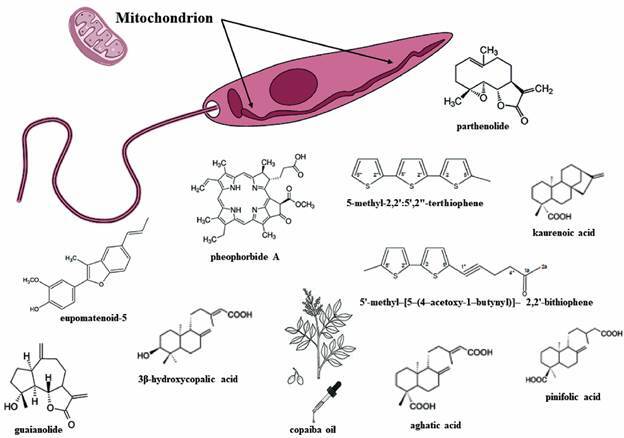
trypanosomatids show unique mitochondrion that is attractive for drug discovery. Major natural antitrypanosomatid compounds evaluated by our research group that cause changes in this organelle.

Consequently, intensive studies of different natural compounds displaying activity against the trypanosomatid mitochondrion unconventional morphology, structure and functionality in contrast with mammalian mitochondria can be useful to understand the mechanism of trypanocidal and leishmanicidal compounds, and can contribute to the design of new and potent therapeutic agents that act with high specificity only in trypanosomatids mitochondrion targets. Multidisciplinary approaches to advancing drug development should be widely encouraged.

Several potential targets can be evaluated in trypanosomatid mitochondrion, such as topoisomerases, kDNA, RNA editing, electron transport chain and associated enzymes. In our perspective, the search of multi-targeted natural compounds in this organelle, able to simultaneously act on two or more mitochondrial targets, is one strategy to improve the efficacy, decrease the toxicity and drug-resistance problems.

In addition, molecular docking and combination therapies are approaches useful for drug discovery against trypanosomatids. Molecular docking analysis is a powerful tool that describes the interaction between small molecules (compound/ligand) with active sites, receptor residues (protein) of interest. This approach is important to identify potential mitochondrial targets for already available drugs and natural products. Molecular docking can also contribute to the development of small molecules from natural compounds which can be taken up by trypanosomatids mitochondrion and act on specific mitochondrial targets. Combination therapies improve the efficacy of the treatment and reduce the risk of resistance. Consequently, more efforts should be deployed to develop new chemotherapeutic strategies using a combination of natural compounds that inhibit different mitochondrial targets of the parasites.

Therefore, in view of the above, we believe that these strategies are essential for the development of an effective chemotherapeutic agent for neglected diseases caused by trypanosomatids.
